# Polymorphism of the Transferrin Gene in Eye Diseases: Keratoconus and Fuchs Endothelial Corneal Dystrophy

**DOI:** 10.1155/2013/247438

**Published:** 2013-11-24

**Authors:** Katarzyna A. Wójcik, Ewelina Synowiec, Manuel P. Jiménez-García, Anna Kaminska, Piotr Polakowski, Janusz Blasiak, Jerzy Szaflik, Jacek P. Szaflik

**Affiliations:** ^1^Department of Molecular Genetics, University of Lodz, Pomorska 141/143, 90-236 Lodz, Poland; ^2^University of Málaga, Avenida Cervantes 2, 29071 Malaga, Spain; ^3^Department of Ophthalmology, Medical University of Warsaw and Samodzielny Publiczny Kliniczny Szpital Okulistyczny, Sierakowskiego 13, 03-710 Warsaw, Poland

## Abstract

Oxidative stress may play a role in the pathogenesis of keratoconus (KC) and Fuchs endothelial corneal dystrophy (FECD). Iron may promote the stress by the Fenton reaction, so its homeostasis should be strictly controlled. Transferrin is essential for iron homeostasis because it transports iron from plasma into cells. The malfunction of transferrin, which may be caused by variation in its gene (*TF*) variation, may contribute to oxidative stress and change KC and FECD risk. To verify this hypothesis we investigated the association between three polymorphisms of the *TF* gene, g.3296G>A (rs8177178), g.3481A>G (rs8177179), and c.–2G>A (rs1130459), and KC and FECD occurrence. Genotyping was performed in blood lymphocytes in 216 patients with KC, 130 patients with FECD and 228 controls by PCR-RFLP. We studied also the influence of other risk factors. The A/A genotype and the A allele of the g.3296G>A polymorphism were associated with KC occurrence, while the G allele was negatively correlated with it. We observed a decrease in KC occurrence associated with the A/G genotype of the g.3481A>G polymorphism. We did not find any association between the c.–2G>A polymorphism and KC. No association was found between all three polymorphisms and FECD occurrence.

## 1. Introduction

Iron is an essential element in most biological systems. It exists in two, ferrous (Fe^2+^) or ferric (Fe^3+^), oxidative states and the exchange between them may play an important role in many biological processes [1]. However, such exchange may catalyze the Fenton reaction, leading to the production of highly reactive hydroxyl radicals (OH^•^) [2]. Reactive oxygen species (ROS), including OH^•^, can react with cellular compounds lipids, proteins, and nucleic acids, altering their structure and functions [3, 4]. Moreover, ROS can cause lipid peroxidation, producing malondialdehyde (MDA) and 4-hydroxy-2-nonenal (HNE), which are highly reactive and have a longer half-life than OH^•^ [5]. Any excess of ROS should be neutralized by antioxidant defense mechanisms, but, under certain circumstances, these mechanisms may not be effective enough, resulting in a net ROS production and oxidative stress [6].

Due to the harmful potential of iron, its metabolism must be strictly regulated both in individual cells and whole organism. Iron overload is involved in numerous diseases, including hemochromatosis and thalassemia [7]. Moreover, a local excess of iron is associated with serious neurological disturbances, Alzheimer's and Parkinson's diseases, which could be caused by an accumulation of iron in areas of the brain [8, 9]. In mammals, ferroportin and divalent metal transporter 1 (DMT1) regulate iron uptake across the duodenal mucosa in response to signals from the liver [10, 11]. In addition, the uptake of iron depends on transferrin (TF) and transferrin receptors (TfR) [12]. TF binds ferric ions and transports them to TfR at the cell surface [13, 14]. Cytosolic iron is regulated also by ferritin and other proteins [15].

Fuchs endothelial corneal dystrophy (FECD) is a slowly progressive disease of the cornea, exhibiting usually symptoms in the fifth or sixth decade of life [16]. The disorder manifests by the formation of excrescences on a thickened Descemet's membrane called *corneal guttae*, progressive loss of corneal endothelial cells, corneal edema, and decreased visual acuity [17]. FECD can be classified into late-onset or early-onset (familial FECD) variants [18]. The pathogenesis of the disease is complex and not known completely and possibly involves interaction between genetic and environmental factors [19]. The early-onset FECD may be associated with mutations in the *COL8A2* gene, encoding the *α*2 subtype of collagen VIII [20], whereas late-onset FECD may be associated with mutations in the *SLC4A11* gene, which encodes a zinc finger transcription factor and the *TCF8* gene encoding sodium borate transporter [21–23]. 

Keratoconus (KC) usually occurs in the teenage years or the second decade of life [24]. The disease is characterized by deformation of the normal shape of the cornea resulting from thinning of the corneal stroma [25]. In most cases KC affects both eyes, inducing irregular astigmatism and myopia [26, 27]. Numerous reports on familial aggregation, twin studies, and genetic analyses indicate the role of genetic compounds in KC pathogenesis [28, 29]. Several genes were proposed as candidates for KC, including visual system homeobox 1 (*VSX1*), superoxide dismutase 1 (*SOD1*), and collagen, type IV, alpha 3 (*COL4A3*) and alpha 4 (*COL4A4*) genes,but their precise role in KC development is not clear [30–32].

Among environmental factors, which may be important in the pathogenesis of KC and FECD, oxidative stress seems to play a special role [33–35]. This is supported by increased levels of oxidative stress markers, including MDA and 8-hydroxy-2′-deoxyguanosine (8-oxoG), as well as downregulation of some antioxidants genes reported in KC and FECD corneas [36, 37].

Because iron may be involved in induction of oxidative stress that plays a role in KC and FECD pathogenesis, we hypothesized that iron disturbances, especially genetic variation in proteins of iron homeostasis, may contribute to development of KC and FECD. In this study we checked the association between three polymorphisms in the *TF* gene, g.3296G>A, g.3481A>G, and c.–2G>A, and the occurrence of FECD and KC. 

## 2. Materials and Methods

### 2.1. Clinical Subjects

The study population was comprised of 216 patients with KC, 130 patients with FECD, and 228 individuals with FECD/KC exclusion (controls). All patients and controls were examined in the Department of Ophthalmology, Medical University of Warsaw (Warsaw, Poland). Medical history was obtained from all subjects and no one reported any genetic disease. 

The diagnosis of KC was based on clinical signs and topographical and pachymetric parameters on TMS corneal topography and Orbscan examinations [27, 38, 39]. The map patterns were carefully interpreted manually in all cases. Patients underwent ophthalmic examination, including best-corrected visual acuity, intraocular pressure, slit lamp examination, fundus examination, corneal topography (TMS4, Tomey, Nagoya, Japan), Orbscan corneal topographical and pachymetrical maps, (Orbscan IIz, Bausch & Lomb, USA). 

The diagnosis of FECD was based on clinical signs on the slit lamp examination (occurrence of endothelial guttae, corneal edema) and in all the cases was confirmed by the presence of specific lesions, polymegathism, and pleomorphism of the endothelial cells in in vivo confocal microscopy (IVCM) examination [40, 41]. Patients underwent ophthalmic examination, including best-corrected visual acuity, intraocular pressure, slit lamp examination, fundus examination, IVCM, and anterior segment optical coherence tomography including pachymetry maps (AS-OCT). The IVCM was performed by a white light scanning slit confocal microscopy system (ConfoScan 3 or ConfoScan 4, Nidek Technologies, Padova, Italy). The AS-OCT was performed by Swept Source Anterior Segment Casia OCT (Tomey, Nagoya, Japan).

The control subjects had no clinical evidence of FECD/KC and presented healthy corneal endothelium on IVCM and normal corneal topography and pachymetry. 

Five milliliters of venous blood was collected from each individual enrolled in this study into EDTA-containing tubes, coded, and stored at −20°C until further use. All participants were interviewed using a structural questionnaire to determine demographic and potential risk factors for KC and FECD. Study cases and controls provided information on their age, lifestyle habits, including smoking, body mass index (BMI), allergy, cooccurrence of visual impairment (hyperopia, astigmatism, and myopia), family history among 1st degree relatives for KC or FECD, and heart or vascular diseases. Smoking was categorized to current, former, or never smokers. Characteristics of patients and controls are presented in Table 1. All individuals employed in our research were unrelated. The study design was approved by the Bioethics Committee of the Medical University of Warsaw and each individual enrolled in this study gave a written informed consent. 

### 2.2. Polymorphism Selection and Primer Design

We searched the public domain of the Single Nucleotide Polymorphism database (dbSNP) at the National Center for Biotechnology Information (NCBI, http://www.ncbi.nlm.nih.gov/snp) and the related literature to identify potentially functional polymorphisms in the *TF* gene. We selected polymorphisms of the known distribution in the European population. SNPs selection favored those with a minor allele frequency not lesser than 3%. Our choice was mainly determined by a potential biological significance of the polymorphisms determined by their location.

Finally, we chose to genotype the g.3296G>A (rs8177178), g.3481A>G (rs8177179), and c.−2G>A (rs1130459) polymorphisms with a minor allele frequency (MAF) 0.342 for rs8177178, 0.422 for rs8177179, and 0.425 for rs1130459 in the European population (submitter population ID: HapMap-CEU; http://www.ncbi.nlm.nih.gov/snp). Primers were designed according to the published nucleotide sequence in ENSEMBL database (gene ID ENSG00000051180) and using Primer3 software (http://frodo.wi.mit.edu/).

### 2.3. DNA Extraction

Genomic DNA was extracted from venous blood by using the commercially available AxyPrep Blood Genomic DNA Miniprep Kit (Axygen Biosciences, Union City, CA, USA), according to the manufacturers instruction. DNA was directly isolated from the white blood cells. DNA purity and concentration were determined by comparing the absorbance at 260 and 280 nm. The purified genomic DNA was stored in TE buffer (5 mM Tris-HCl, 0.1 mM EDTA, pH 8.5), at −20°C until further analysis.

### 2.4. Genotyping

**Figure 1:**
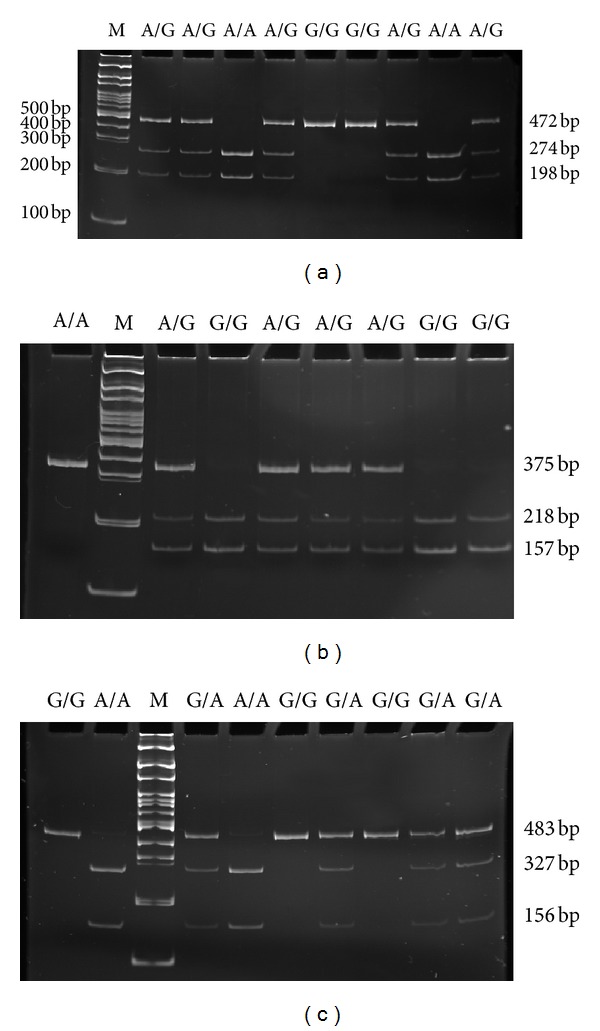
Genotypes of the g.3481A>G (rs8177179) (a), c.−2G>A (rs1130459) (b), and g.3296G>A (rs8177178) (c) polymorphisms of transferrin gene analyzed by 8% polyacrylamide gel electrophoresis stained with ethidium bromide and viewed under UV light. Lane M shows 100 bp Ladder DNA molecular weight marker.

The genotypes of the polymorphisms were determined by the polymerase chain reaction-restriction fragment length polymorphism (PCR-RFLP) method. DNA was amplified in a final reaction volume of 10 *μ*L including 25 ng of genomic DNA, 5 *μ*L 2 × KAPA Taq Ready Mix containing KapaTaq DNA polymerase (0.025 U/*μ*L), Reaction Buffer with MgCl_2_ and 0.2 mM each dNTP (Kapa Biosystems, Woburn, MA, USA), and 0.25 *μ*M of each primer (Sigma-Aldrich, St. Louis, MO, USA). The following primers were used: for g.3296G>A: forward 5′-AGGGCATAGAGCTGGCTGCT-3′, reverse 5′-GAAGACTGTTAGCATGAGGGCC-3′; for g.3481A>G: forward 5′-AGCTGTATGTGTGCATGCTGCTC-3′, reverse 5′-GGGCCAATTCACACATTCAAT-3; for c.–2G>A: forward 5′-AGAAAATGAGGTGATCAGTGGG-3′, reverse 5′-ATGGAAAGGCACCCAGACAC-3. PCR assay was performed in a C1000 Thermal Cycler (Bio-Rad, Hercules, CA, USA) under the following conditions: initial denaturation step at 95°C for 5 min, 32 cycles of denaturation at 95°C for 30 s, annealing for 30 s at temperature dependent on used primers, amplification at 72°C for 1 min, and final extension at 72°C for 5 min. Amplified fragments containing the g.3296G>A, g.3481A>G, and c.–G>A polymorphic sites were digested by the *Rsa*I, *Sty*I, and *Fok*I restrictases (New England Biolabs, Ipswich, UK), respectively. The digestion of 5 *μ*L of PCR product was performed with 2 U of respective restriction enzyme in a total volume of 15 *μ*L for 16 h at 37°C. The length of restriction products and genotypes are shown in Table 2. After digestion, products were separated on an 8% polyacrylamide gel. Electrophoresis was carried out at 5 V/cm in TBE buffer. A 100 bp Ladder DNA (Axygen Biosciences) was used as a molecular mass marker. After separation, gels were stained with ethidium bromide (0.5 mg/mL) and viewed in UV light. Gels were analyzed with using the digital imaging system InGenius Bio Imaging (Syngene, Cambridge, UK). Representative gels from the analysis of these polymorphisms are presented in Figure 1. 

### 2.5. Statistical Analysis

Statistical analyses were carried out with the SigmaPlot software, version 11.0 (Systat Software, Inc., San Jose, CA, USA). To compare the distributions of demographic and potential risk factors between patients and controls, the Chi-square (*χ* ^2^) test was used. The Hardy-Weinberg equilibrium was checked using *χ* ^2^ test to compare the observed and expected genotype frequencies. The *χ* ^2^ analysis was also used to test the significance of the differences between distributions of genotypes and alleles in KC/FECD patients and controls. The association between case-control status and each polymorphism, measured by the odds ratio (OR) and its corresponding 95% confidence interval (CI), was estimated using an unconditional multiple logistic regression model, both with and without adjustment for age, sex, co-occurrence of visual disturbances, smoking, and family status of KC/FECD. 

## 3. Results

### 3.1. Characteristics of the Study Subjects

Demographic variables and potential risk factors for KC and FECD of the patients and controls are shown in Table 1. We observed a significant difference in age between KC/FECD patients and controls. However, as the mean age for control was almost 62 years, we were almost sure that this group did not contain cases, which might develop KC, as it is a disease observed in much younger individuals. Further analysis for FECD was adjusted for age. There was a difference in sex distribution between KC patients and controls and sex was taken into account as a confounding factor in further analysis. No difference was observed between sex distributions for FECD patients and controls, which is important as a clear shift toward women was reported in several studies on FECD epidemiology [17]. We observed significant differences between distribution of family history (1st degree relatives) for KC (positive versus negative family history), allergies (yes versus never) heart or vascular diseases (yes versus never), and cooccurrence of visual impairment (yes versus no) among KC patients and controls. In addition, we showed significant differences between distribution of family history (1st degree relatives) for FECD (positive versus negative family history), heart or vascular diseases (yes versus never), and cooccurrence of visual impairment (yes versus no) among FECD patients and controls. These parameters were further adjusted in the multivariate logistic regression model for possible confounding factors of the main effect of the SNPs. 

### 3.2. The g.3296G>A, g.3481A>G, and c.–2G>A Polymorphisms of the *TF* Gene and KC Occurrence

The genotype and allele distributions of the g.3296G>A, g.3481A>G, and c.−2G>A polymorphisms of the *TF* gene in KC patients and controls are presented in Table 3. The observed genotypes frequencies did not differ significantly from the Hardy-Weinberg equilibrium (*P* > 0.05, data not shown) for each group. We observed a significant (*P* < 0.05) difference in the frequency of distributions of genotypes of the g.3296G>A polymorphism between the cases and controls. The presence of the A/A genotype and A allele of the g.3296G>A polymorphism significantly increased the occurrence of KC. On the other hand, the G allele decreased it. We also observed a decrease in KC occurrence associated with the A/G genotype of the g.3481A>G polymorphism. We did not find any association between genotypes/alleles of the c.−2G>A polymorphism and KC occurrence.

### 3.3. The g.3296G>A, g.3481A>G, and c.−2G>A Polymorphisms of the *TF* Gene and FECD Occurrence

The genotype and allele distributions of the g.3296G>A, g.3481A>G, and c.-2G>A polymorphisms in FECD patients and controls are presented in Table 4. There was not any difference in the frequency of distributions of genotypes of these polymorphisms between patients and controls (*P* > 0.05) and the observed genotype frequencies did not differ from the Hardy-Weinberg equilibrium (*P* > 0.05; data not shown). We did not observe any association between genotypes/alleles of these polymorphisms and FECD occurrence. 

## 4. Discussion

A growing body of evidence indicates a role of oxidative stress in KC and FECD pathogenesis [34, 38, 42]. Increased levels of nitrotyrosine, a product of nitric oxide metabolism, inducible nitric oxide synthase (iNOS), and MDA were reported in KC and FECD corneas, compared to normal corneas [36]. In addition, a downregulation of some oxidative-stress-related genes in corneas of both disorders was observed [35, 42]. FECD endothelial cells exhibited a decreased expression and/or activity of peroxiredoxins, thioredoxin reductase, metallothionein 3, glutathione S-transferase, superoxide dismutase 2, ferritin, and heat shock 70 kDa protein [35, 43]. Moreover, FECD endothelium showed a decreased level of the Nrf2 protein, which is involved in transcriptional activation of genes encoding proteins important for the protection against oxidative stress [35, 44]. Keratoconus corneas showed a deficiency in aldehyde dehydrogenase class 3 (ALDH3) and a decreased activity of extracellular superoxide dismutase (EC-SOD) compared to controls [33, 45]. Also, KC corneas were characterized by deposition of iron at the level of the epithelial basement membrane; thus this tissue may be more susceptible to oxidative stress [23]. Furthermore, both diseases were characterized by an increased level of mitochondrial DNA (mtDNA) damage as compared with age-matched controls [35, 46] and abnormal levels of some proteins of oxidative phosphorylation encoded by mtDNA [34, 46]. Due to the close vicinity of the ROS-producing respiratory chain, mtDNA is especially prone to oxidative damage, which may lead to defective functioning of the chain proteins, causing accumulation of ROS and increasing the extent of oxidative stress. Despite our growing knowledge of the role of oxidative stress in KC and FECD pathogenesis, basic factor(s) underlying these diseases is not clear. In this work we suggest that variation in gene encoding of a major protein of iron homeostasis may influence KC and FECD susceptibility.

Though iron is essential for a number of cellular functions, capability of free iron to generate free radicals *via* the Fenton reaction may be associated with induction of oxidative stress [10]. Results of numerous studies suggest that intraocular iron may contribute to a broad range of ocular diseases. Retinal degeneration resulting from iron overload was found in hereditary disorders including aceruloplasminemia, Friedreich's ataxia, and Hallervorden-Spatz disease [47]. Therefore, the level of intracellular iron may be influenced by various mechanisms [48]. 

Transferrin is one of the major serum proteins that plays an important role in the maintaining of iron homeostasis as well as the prevention of oxidative damage induced by free radicals [1]. Transferrin is a glycoprotein that controls the levels of free iron in body fluids by binding and transporting ferric ions [7]. In addition, TF is involved in transport of a wide range of other divalent, trivalent, and tetravalent metal ions, such as aluminum, zinc, and gallium [48]. Transferrin loaded with iron binds to TfR at the surface of the cell and regulates the uptake and storage of iron [49, 50]. The number of TfR on the plasma membrane is adjusted to iron requirement in the cell [13]. Any disturbance in level or activity of TF may lead to disturbances in iron homeostasis [1]. Moreover, TF is required for cell growth, proliferation, and differentiation [51]. Because TF is one of the serum factors necessary for epithelial cell growth, including corneal epithelial cells, variation in its gene may contribute to corneal epithelium disorders [51]. 

We recently showed that transferrin level was associated with age-related macular degeneration (AMD) [52]. This disease, like FECD, is an eye disease occurring mostly after the fifth decade of life. The pathogenesis of AMD is not completely known, but many genetic and environmental factors are involved in the development of this disease. Numerous results indicated that AMD pathogenesis, just as KC and FECD, is associated with oxidative stress [53, 54]. A growing body of evidence suggests a link between progressive degeneration of the retina in AMD and iron accumulation [55]. Increased iron levels were found in retinas of patients with AMD [54]. Furthermore, we showed that the g.3296G>A and c.–576G>A polymorphisms of the *TF* gene may be correlated with the increased risk of developing AMD [52]. Transferrin binds free iron ions; thus it may protect the tissue from potentially toxic effects of unbound iron [51]. An association between genetic variability in the *TF* gene and increased oxidative stress, resulting in degeneration of the retina in AMD, suggests a potentially significant role of transferrin in the eye [56, 57]. These data suggest that variation in the *TF *gene may contribute to increased level of unbound iron, resulting in oxidative injury and eye disorder.

To our knowledge, this is the first report describing a role of the *TF* gene in corneal diseases. In the study, we investigated the influence of three polymorphisms in the *TF* gene on the prevalence of KC and FECD in the population of central and eastern Poland. Two of these polymorphisms are located in the 5′ flanking region of these genes and one in 5′-UTR. The role of these polymorphisms is not known, but their locations suggest a possible effect on gene expression level. Our reports revealed that the occurrence of the A/A genotype and the A allele of the g.3296G>A polymorphism of the *TF* gene was associated with increased occurrence of KC. Moreover, we observed that the presence of the G allele of the g.3296G>A polymorphism of the *TF* gene decreased KC occurrence. Variation in the *TF* gene may influence its protein content or activity, resulting in iron accumulation which was showed in KC cornea and increased susceptibility to oxidative injury. We did not find any association between the c.–2G>A polymorphism and KC. Moreover, we found no association between the 3296G>A, g.3481A>G, and c.–2G>A polymorphisms and FECD occurrence. 

Results of the genome-wide association study conducted on European and Asian populations detected multiple loci associated with central corneal thickness, the main clinical feature of KC [58, 59]. These regions contain the *COL5A1*, *AVGR8*, *FOXO1*, *AKAP13*, and *ZNF469* genes but do not include the *TF* gene [59–63]. However, our study, the first searching for the association between KC and variability of the *TF* gene, needs replication in other subpopulation(s), before drawing a definite conclusion on a new locus of KC susceptibility. On the other hand, central corneal thickness is a KC clinical feature, which does not have to be underlined by all genes involved in KC pathogenesis.

Genome-wide linkage analyses identified multiple chromosomal risk regions for FECD, including 5q33.1–q35.2, 9p22.1–9p24.1, 13ptel–13q12.13, and 18q21.2–q21.32 [21, 64–67]. Moreover, genome-wide association study on the European population showed only two regions in the transcription factor 4 (*TCF4*) gene as associated with FECD [68]. Lack of the *TF* gene in these regions and no association polymorphism mutations in *TF* locus and FECD susceptibility detected in our studies may suggest that it may not be involved in FECD pathogenesis in the subpopulation under study.

## 5. Conclusion

The g.3296G>A, g.3481A>G, c.–2G>A and polymorphisms of the *TF* gene may modulate the risk of keratoconus, by its involvement in altered iron homoeostasis and oxidative stress induction. 

## Figures and Tables

**Table 1:** Characteristics of keratoconus (KC) and Fuchs endothelial corneal dystrophy (FECD) patients and controls enrolled in this study.

Feature	Controls (*n* = 228)		KC (*n* = 216)		*P *	FECD (*n* = 130)		*P *
	Number	Frequency	Number	Frequency		Number	Frequency	
Sex								
Females	148	0.65	66	0.31	**<0.001**	93	0.72	0.20
Males	80	0.35	150	0.69		37	0.28	
Age								
Mean ± SD	61.92 ± 19.77		36.24 ± 11.78		<0.001*	70.00 ± 10		<0.001*
Range	19–100		14–63			37–91		
Smoking								
Yes (current/former)	84	0.42	69	0.32	0.324	42	0.32	0.453
Never	144	0.58	147	0.68		88	0.68	
KC/FECD in family								
Yes	9	0.04	26	0.12	**0.0028**	13	0.10	**0.039**
No	219	0.96	190	0.88		117	0.90	
BMI								
≤25	99	0.44	97	0.45	0.986	51	0.40	0.513
25–30	74	0.33	71	0.33		41	0.32	
≥30	51	0.23	48	0.22		36	0.28	
Visual impairment								
Yes	150	0.66	158	0.73	0.114	129	0.99	**<0.001**
No	78	0.34	58	0.27		1	0.01	
Allergies								
Yes	33	0.14	62	0.29	**0.0004**	21	0.17	0.784
No	195	0.86	154	0.71		109	0.83	
Heart and vascular diseases								
Yes	115	0.50	46	0.21	**<0.001**	82	0.63	**0.028**
No	113	0.50	170	0.79		48	0.37	

*P* values for two-side *χ* ^2^ test, except *P values for *t*-test; *P* < 0.05 are in bold.

**Table 2:** Data for PCR-RFLP analysis used to genotype polymorphisms in the *TF* gene.

Polymorphism	Primers sequences (forward, reverse)	Annealing temperatures in PCR (°C)	Restriction enzymes (recognized allele)	Fragments (bp)
g.3296G>A rs8177178	AGGGCATAGAGCTGGCTGCT GAAGACTGTTAGCATGAGGGCC	66.2	*Rsa*I (A)	GG 483 GA 483/327/156 AA 327/156
g.3481A>G rs8177179	AGCTGTATGTGTGCATGCTGCTC GGGCCAATTCACACATTCAAT	60.5	*Sty*I (A)	AA 274/198 AG 472/274/198 GG 472
c.–2G>A rs1130459	AGAAAATGAGGTGATCAGTGGG ATGGAAAGGCACCCAGACAC	66.0	*Fok*I (G)	GG 218/157 GA 375/218/157 AA 375

**Table 3:** Distribution of genotypes and alleles of the g.3296G>A, g.3481A>G, and c.–2G>A polymorphisms of the *TF* gene and odds ratio (OR) with 95% confidence interval (95% CI) in patients with keratoconus (KC) and controls.

Polymorphism Genotype/allele	Controls (*n* = 228)		KC (*n* = 216)		Crude OR (95% CI)	*P *	Adjusted OR (95% CI)	*P *
	Number	Frequency	Number	Frequency				
g.3296G>A								
G/G	103	0.45	82	0.38	0.74 (0.50–1.08)	0.114	0.64 (0.42–1.00)	0.048
G/A	115	0.50	106	0.49	0.96 (0.66–1.39)	0.809	1.09 (0.71–1.66)	0.708
A/A	10	0.04	28	0.13	**3.23 (1.53–6.83)**	**0.002**	**3.22 (1.40–7.42)**	**0.006**
*χ* ^2^ = 10.935; *P* = 0.004								
G	320	0.70	270	0.62	**0.68 (0.50–0.91)**	**0.011**	**0.62 (0.44–0.88)**	**0.007**
A	134	0.30	162	0.38	**1.52 (1.12–2.05)**	**0.007**	**1.66 (1.17–2.35)**	**0.005**

g.3481A>G								
A/A	62	0.27	68	0.31	1.23 (0.82–1.85)	0.321	1.34 (0.84–2.13)	0.225
A/G	132	0.58	106	0.49	0.70 (0.48–1.02)	0.063	**0.64 (0.42–0.99)**	**0.043**
G/G	34	0.15	42	0.19	1.38 (0.84–2.26)	0.206	1.41 (0.80–2.48)	0.230
*χ* ^2^ = 3.638; *P* = 0.162								
A	256	0.56	242	0.56	0.99 (0.75–1.31)	0.969	1.02 (0.75–1.40)	0.885
G	200	0.44	190	0.44	1.00 (0.76–1.33)	0.969	0.98 (0.72–1.33)	0.885

c.–2G>A								
G/G	61	0.27	68	0.31	1.23 (0.82–1.84)	0.327	1.31 (0.82–2.07)	0.257
G/A	128	0.56	104	0.48	0.73 (0.50–1.05)	0.092	0.69 (0.45–1.05)	0.084
A/A	39	0.17	44	0.20	1.24 (0.77–1.99)	0.378	1.23 (0.72–2.12)	0.451
*χ* ^2^ = 2.683; *P* = 0.261								
G	250	0.55	240	0.56	1.05 (0.80–1.38)	0.719	1.09 (0.80–1.48)	0.605
A	206	0.45	192	0.44	0.97 (0.74–1.27)	0.822	0.94 (0.69–1.28)	0.693

*P* < 0.05 along with corresponding ORs are in bold; ^a^OR adjusted for heart and vascular diseases, allergies, sex, and family history for KC.

**Table 4:** Distribution of genotypes and alleles of the g.3296G>A, g.3481A>G, and c.–2G>A polymorphisms of the *TF* gene and odds ratio (OR) with 95% confidence interval (95% CI) in patients with Fuchs endothelial corneal dystrophy (FECD) and controls.

Polymorphisms Genotype/allele	Controls (*n* = 228)		FECD (*n* = 130)		Crude OR (95% CI)	*P *	Adjusted OR (95% CI)	*P *
	Number	Frequency	Number	Frequency				
g.3296G>A								
G/G	103	0.45	50	0.38	0.75 (0.49–1.17)	0.205	0.82 (0.50–1.34)	0.420
G/A	115	0.50	71	0.55	1.19 (0.77–1.84)	0.424	1.11 (0.68–1.82)	0.666
A/A	10	0.04	9	0.07	1.61 (0.64–4.08)	0.312	1.58 (0.52–4.79)	0.418
*χ* ^2^ = 2.214; *P* = 0.331								
G	320	0.70	171	0.66	0.78 (0.54–1.12)	0.173	0.82 (0.54–1.25)	0.356
A	134	0.30	89	0.34	1.33 (0.92–1.92)	0.132	1.25 (0.82–1.91)	0.302

g.3481A>G								
A/A	62	0.27	35	0.27	0.99 (0.61–1.60)	0.956	0.88 (0.51–1.53)	0.661
A/G	132	0.58	71	0.55	0.88 (0.57–1.35)	0.547	1.02 (0.62–1.66)	0.902
G/G	34	0.15	24	0.18	1.29 (0.73–2.29)	0.382	1.15 (0.60–2.18)	0.676
*χ* ^2^ = 0.803; *P* = 0.669								
A	256	0.56	141	0.54	0.91 (0.66–1.27)	0.592	0.90 (0.63–1.31)	0.592
G	200	0.44	119	0.46	1.09 (0.79–1.53)	0.592	1.11 (0.77–1.60)	0.592

c.–2G>A								
G/G	61	0.27	35	0.27	0.99 (0.61–1.59)	0.957	0.87 (0.52–1.44)	0.596
G/A	128	0.56	71	0.55	0.94 (0.61–1.45)	0.780	0.99 (0.60–1.61)	0.958
A/A	39	0.17	24	0.18	1.09 (0.63–1.92)	0.746	1.15 (0.60–2.18)	0.676
*χ* ^2^ = 0.122; *P* = 0.941								
G	250	0.55	141	0.54	0.99 (0.72–1.38)	0.966	0.95 (0.66–1.38)	0.799
A	206	0.45	119	0.46	1.03 (0.74–1.42)	0.870	1.09 (0.75–1.57)	0.658

^a^OR adjusted for cooccurrence of visual impairment, heart and vascular diseases, age, and family history for FECD.

## References

[1] Lieu PT, Heiskala M, Peterson PA, Yang Y (2001). The roles of iron in health and disease. *Molecular Aspects of Medicine*.

[2] Andrews NC (1999). Disorders of iron metabolism. *New England Journal of Medicine*.

[3] Dröge W (2002). Free radicals in the physiological control of cell function. *Physiological Reviews*.

[4] Kryston TB, Georgiev AB, Pissis P, Georgakilas AG (2011). Role of oxidative stress and DNA damage in human carcinogenesis. *Mutation Research*.

[5] Uchida K (2000). Role of reactive aldehyde in cardiovascular diseases. *Free Radical Biology and Medicine*.

[6] Sies H (1997). Oxidative stress: oxidants and antioxidants. *Experimental Physiology*.

[7] Sheftel AD, Mason AB, Ponka P (2012). The long history of iron in the Universe and in health and disease. *Biochimica et Biophysica Acta*.

[8] Ong WY, Tanaka K, Dawe GS, Ittner LM, Farooqui AA (2013). Slow excitotoxicity in Alzheimer's disease. *Journal of Alzheimer's Disease*.

[9] Weinreb O, Mandel S, Youdim MB, Amit T (2013). Targeting dysregulation of brain iron homeostasis in Parkinson's disease by iron chelators. *Free Radical Biology & Medicine*.

[10] Rouault TA (2006). The role of iron regulatory proteins in mammalian iron homeostasis and disease. *Nature Chemical Biology*.

[11] Hentze MW, Muckenthaler MU, Andrews NC (2004). Balancing acts: molecular control of mammalian iron metabolism. *Cell*.

[12] Ponka P, Lok CN (1999). The transferrin receptor: role in health and disease. *International Journal of Biochemistry and Cell Biology*.

[13] Li H, Qian ZM (2002). Transferrin/transferrin receptor-mediated drug delivery. *Medicinal Research Reviews*.

[14] Richardson DR, Dickson L, Baker E (1996). Intermediate steps in cellular iron uptake from transferrin. II. A cytoplasmic pool of iron is released from cultured cells via temperature- dependent mechanical wounding. *In Vitro Cellular and Developmental Biology*.

[15] Harrison PM, Arosio P (1996). The ferritins: molecular properties, iron storage function and cellular regulation. *Biochimica et Biophysica Acta*.

[16] Eghrari AO, Gottsch JD (2010). Fuchs corneal dystrophy. *Expert Review of Ophthalmology*.

[17] Elhalis H, Azizi B, Jurkunas UV (2010). Fuchs endothelial corneal dystrophy. *Ocular Surface*.

[18] Thalamuthu A, Khor CC, Venkataraman D (2011). Association of TCF4 gene polymorphisms with Fuchs’ corneal dystrophy in the Chinese. *Investigative Ophthalmology and Visual Science*.

[19] Schmedt T, Silva MM, Ziaei A, Jurkunas U (2012). Molecular bases of corneal endothelial dystrophies. *Experimental Eye Research*.

[20] Hemadevi B, Srinivasan M, Arunkumar J, Prajna NV, Sundaresan P (2010). Genetic analysis of patients with Fuchs endothelial corneal dystrophy in India. *BMC Ophthalmology*.

[21] Riazuddin SA, Zaghloul NA, Al-Saif A (2010). Missense mutations in TCF8 cause late-onset Fuchs corneal dystrophy and interact with FCD4 on chromosome 9p. *American Journal of Human Genetics*.

[22] Vithana EN, Morgan PE, Ramprasad V (2008). SLC4A11 mutations in Fuchs endothelial corneal dystrophy. *Human Molecular Genetics*.

[23] Riazuddin SA, Vithana EN, Seet L (2010). Missense mutations in the sodium borate cotransporter SLC4A11 cause late-onset Fuchs corneal dystrophya. *Human Mutation*.

[24] Romero-Jiménez M, Santodomingo-Rubido J, Wolffsohn JS (2010). Keratoconus: a review. *Contact Lens and Anterior Eye*.

[25] Sherwin T, Brookes NH (2004). Morphological changes in keratoconus: pathology or pathogenesis. *Clinical and Experimental Ophthalmology*.

[26] Timucin OB, Karadag MF, Cinal A (2013). Assessment of keratocyte density in patients with keratoconus not using contact lenses. *Contact Lens and Anterior Eye*.

[27] Rabinowitz YS (1998). Keratoconus. *Survey of Ophthalmology*.

[28] Edwards M, McGhee CNJ, Dean S (2001). The genetics of keratoconus. *Clinical and Experimental Ophthalmology*.

[29] Nielsen K, Hjortdal J, Pihlmann M, Corydon TJ (2013). Update on the keratoconus genetics. *Acta Ophthalmologica*.

[30] Tanwar M, Kumar M, Nayak B (2010). VSX1 gene analysis in keratoconus. *Molecular Vision*.

[31] Stabuc-Silih M, Ravnik-Glavac M, Glavac D, Hawlina M, Strazisar M (2009). Polymorphisms in COL4A3 and COL4A4 genes associated with keratoconus. *Molecular Vision*.

[32] Stabuc-Silih M, Strazisar M, Ravnik-Glavac M, Hawlina M, Glavac D (2010). Genetics and clinical characteristics of keratoconus. *Acta Dermatovenerologica Alpina*.

[33] Kenney MC, Brown DJ (2003). The cascade hypothesis of keratoconus. *Contact Lens and Anterior Eye*.

[34] Chwa M, Atilano SR, Hertzog D (2008). Hypersensitive response to oxidative stress in keratoconus corneal fibroblasts. *Investigative Ophthalmology and Visual Science*.

[35] Jurkunas UV, Bitar MS, Funaki T, Azizi B (2010). Evidence of oxidative stress in the pathogenesis of fuchs endothelial corneal dystrophy. *American Journal of Pathology*.

[36] Buddi R, Lin B, Atilano SR, Zorapapel NC, Kenney MC, Brown DJ (2002). Evidence of oxidative stress in human corneal diseases. *Journal of Histochemistry and Cytochemistry*.

[37] Arnal E, Peris-Martínez C, Menezo JL, Johnsen-Soriano S, Romero FJ (2011). Oxidative stress in keratoconus?. *Investigative Ophthalmology & Visual Science*.

[38] Holladay JT (2009). Keratoconus detection using corneal topography. *Journal of Refractive Surgery*.

[39] Pflugfelder SC, Liu Z, Feuer W, Verm A (2002). Corneal thickness indices discriminate between keratoconus and contact lens-induced corneal thinning. *Ophthalmology*.

[40] Szaflik JP (2007). Comparison of in vivo confocal microscopy of human cornea by white light scanning slit and laser scanning systems. *Cornea*.

[41] Weiss JS, Møller HU, Lisch W (2008). The IC3D classification of the corneal dystrophies. *Cornea*.

[42] Kenney MC, Chwa M, Atilano SR (2005). Increased levels of catalase and cathepsin V/l2 but decreased TIMP-1 in keratoconus corneas: evidence that oxidative stress plays a role in this disorder. *Investigative Ophthalmology and Visual Science*.

[43] Gottsch JD, Bowers AL, Margulies EH (2003). Serial analysis of gene expression in the corneal endothelium of Fuchs’ dystrophy. *Investigative Ophthalmology and Visual Science*.

[44] Lee J, Calkins MJ, Chan K, Kan YW, Johnson JA (2003). Identification of the NF-E2-related factor-2-dependent genes conferring protection against oxidative stress in primary cortical astrocytes using oligonucleotide microarray analysis. *Journal of Biological Chemistry*.

[45] Behndig A, Karlsson K, Johansson BO, Brännström T, Marklund SL (2001). Superoxide dismutase isoenzymes in the normal and diseased human cornea. *Investigative Ophthalmology and Visual Science*.

[46] Atilano SR, Coskun P, Chwa M (2005). Accumulation of mitochondrial DNA damage in keratoconus corneas. *Investigative Ophthalmology and Visual Science*.

[47] He X, Hahn P, Iacovelli J (2007). Iron homeostasis and toxicity in retinal degeneration. *Progress in Retinal and Eye Research*.

[48] Benkovic SA, Connor JR (1993). Ferritin, transferrin, and iron in selected regions of the adult and aged rat brain. *Journal of Comparative Neurology*.

[49] Aisen P (1992). Entry of iron into cells: a new role for the transferrin receptor in modulating iron release from transferrin. *Annals of Neurology*.

[50] Aisen P (2004). Transferrin receptor 1. *International Journal of Biochemistry and Cell Biology*.

[51] Baudouin C, Brignole F, Fredj-Reygrobellet D, Negre F, Bayle J, Gastaud P (1992). Transferrin receptor expression by retinal pigment epithelial cells in proliferative vitreoretinopathy. *Investigative Ophthalmology and Visual Science*.

[52] Wysokinski D, Danisz K, Blasiak J (2013). An association of transferrin gene polymorphism and serum transferrin levels with age-related macular degeneration. *Experimental Eye Research*.

[53] Beatty S, Koh H, Phil M, Henson D, Boulton M (2000). The role of oxidative stress in the pathogenesis of age-related macular degeneration. *Survey of Ophthalmology*.

[54] Hahn P, Milam AH, Dunaief JL (2003). Maculas affected by age-related macular degeneration contain increased chelatable iron in the retinal pigment epithelium and Bruch’s membrane. *Archives of Ophthalmology*.

[55] Chowers I, Wong R, Dentchev T (2006). The iron carrier transferrin is upregulated in retinas from patients with age-related macular degeneration. *Investigative Ophthalmology and Visual Science*.

[56] Blasiak J, Szaflik J, Szaflik JP (2011). Implications of altered iron homeostasis for age-related macular degeneration. *Frontiers in Bioscience*.

[57] Imamura Y, Noda S, Hashizume K (2006). Drusen, choroidal neovascularization, and retinal pigment epithelium dysfunction in SOD1-deficient mice: a model of age-related macular degeneration. *Proceedings of the National Academy of Sciences of the United States of America*.

[58] Wheeler J, Hauser MA, Afshari NA, Allingham RR, Liu Y (2012). The genetics of keratoconus: a review. *Reproductive System & Sexual Disorders*.

[59] Lu Y, Vitart V, Burdon KP (2013). Genome-wide assiation analyses identify multiple loci associated with central corneal thickness and keratoconus. *Nature Genetics*.

[60] Abu A, Frydman M, Marek D (2008). Deleterious mutations in the zinc-finger 469 gene cause brittle cornea syndrome. *American Journal of Human Genetics*.

[61] Khan AO, Aldahmesh MA, Mohamed JN, Alkuraya FS (2010). Blue sclera with and without corneal fragility (brittle cornea syndrome) in a consanguineous family harboring ZNF469 mutation (p.E1392X). *Archives of Ophthalmology*.

[62] Segev F, Héon E, Cole WG (2006). Structural abnormalities of the cornea and lid resulting from collagen V mutations. *Investigative Ophthalmology and Visual Science*.

[63] Gottsch JD, Sundin OH, Liu SH (2005). Inheritance of a novel COL8A2 mutation defines a distinct early-onset subtype of fuchs corneal dystrophy. *Investigative Ophthalmology and Visual Science*.

[64] Sundin OH, Broman KW, Chang HH, Vito ECL, Stark WJ, Gottsch JD (2006). A common locus for late-onset Fuchs corneal dystrophy maps to 18q21.2-q21.32. *Investigative Ophthalmology and Visual Science*.

[65] Riazuddin SA, Eghrari AO, Al-Saif A (2009). Linkage of a mild late-onset phenotype of fuchs corneal dystrophy to a novel locus at 5q33.1-q35.2. *Investigative Ophthalmology and Visual Science*.

[66] Sundin OH, Jun AS, Broman KW (2006). Linkage of late-onset fuchs corneal dystrophy to a novel locus at 13pTel-13q12.13. *Investigative Ophthalmology and Visual Science*.

[67] Afshari NA, Li Y, Pericak-Vance MA, Gregory S, Klintworth GK (2009). Genome-wide linkage scan in fuchs endothelial corneal dystrophy. *Investigative Ophthalmology and Visual Science*.

[68] Baratz KH, Tosakulwong N, Ryu E (2010). E2-2 protein and Fuchs’s corneal dystrophy. *New England Journal of Medicine*.

